# Material-Dependent Effect of Common Printing Parameters on Residual Stress and Warpage Deformation in 3D Printing: A Comprehensive Finite Element Analysis Study

**DOI:** 10.3390/polym15132893

**Published:** 2023-06-29

**Authors:** Hussein Alzyod, Peter Ficzere

**Affiliations:** Department of Railway Vehicles and Vehicle System Analysis, Faculty of Transportation Engineering and Vehicle Engineering, Budapest University of Technology and Economics, Műegyetem rkp.3, H-1111 Budapest, Hungary; ficzere.peter@kjk.bme.hu

**Keywords:** residual stress, warpage deformation, fused filament fabrication, optimization, finite element analysis, Taguchi method, ANOVA

## Abstract

Additive manufacturing (AM), commonly known as 3D printing, has gained significant popularity for its ability to produce intricate parts with high precision. However, the presence of residual stresses and warpage deformation are common issues affecting the quality and functionality of 3D-printed parts. This study conducts a comprehensive finite element analysis (FEA) to investigate the material-dependent impact of key printing parameters on residual stress and warpage deformation in 3D printing. The research focuses on three distinct materials: polyetherimide (PEI), acrylonitrile butadiene styrene (ABS), and polyamide 6 (PA6). Various printing parameters are systematically varied, including printing temperature, printing speed, bed temperature, infill density, layer thickness, and infill pattern. The study employs the Taguchi L27 orthogonal array and employs the analysis of variance (ANOVA) statistical technique to assess the significance of the input parameters. The obtained results reveal that certain parameters exhibit a greater sensitivity to material differences, whereas the layer thickness parameter demonstrates a relatively lower sensitivity. Notably, infill density and printing temperature play a crucial role in reducing residual stress for PA6, while the infill pattern parameter proves to be a significant contributor to minimizing warpage deformation across all three materials. These findings underscore the importance of conducting material-specific analyses to optimize 3D printing parameters and achieve the desired quality outcomes while mitigating residual stress and warpage deformation.

## 1. Introduction

**Figure 1:**
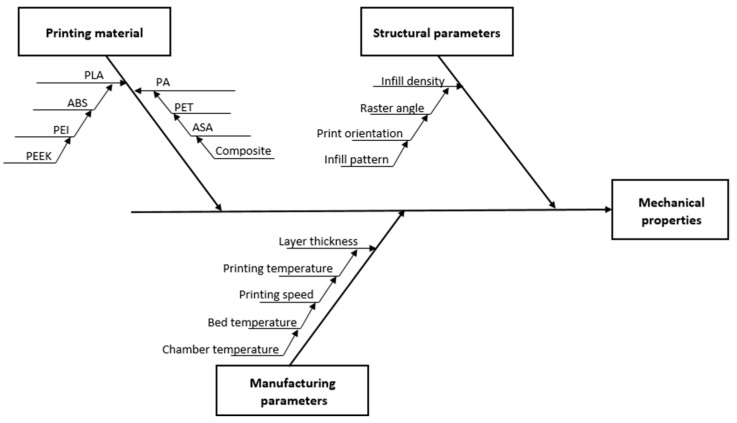
Ishikawa diagram of FFF parameters.

AM, also known as a 3D printing technology, was invented by Charles Hull in the mid-1980s [1]. He defined it at that time as stereolithography (SLA). Its principle is based on building a solid object by successively printing thin layers of material one on top of the other. Nowadays, it is implemented in various sectors of the industry, including automotive [2], aerospace [3], medical [4,5], construction [6], and even food [7]. According to the ASTM standard, AM manufacturing processes are categorized into seven main categories: binder jetting (BJT), directed energy deposition (DED), material extrusion (MEX), material jetting (MJT), powder bed fusion (PBF), sheet lamination (SHL), and VAT photopolymerization (VPP) [8]. In the MEX category, the main 3D printing process is the fused filament fabrication (FFF), or fused deposition filament (FDM), which has been trademarked by Stratasys since 1991 [9]. The use of polymer AM in the market is expected to achieve more than USD 55 billion by 2030 [10]. Utilizing FFF technology presents numerous benefits, including cost-effectiveness, material diversity, printing parts with superior properties, low maintenance requirements, implementation without any hazardous or toxic components, and the ability to produce complex geometries [11]. On the other hand, FFF is still subject to certain limitations, such as restricted printing size, reliance on support structures, prolonged building time, limited resolution, and inadequate precision technology. FFF also has printing and structural parameters, which can be defined in the slicing process, and Figure 1 shows these parameters. The slicing can be performed using software called a slicer.

Printing parameters significantly influence the mechanical properties of printed parts. In order to enhance functionality and expand the range of applications for parts manufactured through additive technology, researchers have been working to optimize the printing parameters [12,13,14,15]. A crucial aspect that affects the mechanical performance of parts produced through AM is the repeated heating and cooling cycles during the layer-by-layer construction process [16,17]. These cycles can lead to the formation of residual stresses, which can have a negative impact on mechanical performance and result in distortion and inaccuracies in the dimensions of the part [18,19,20,21]. Researchers have made numerous experimental efforts to characterize residual stresses and warpage deformation in additively manufactured parts. For instance, Karalekas and Rapti [22] utilized an epoxy-based photopolymer to study the relationship between residual stress and processing in stereolithography (SLA) solidification using the hole-drilling strain-gage method for stress relaxation. In another study, Karalekas and Aggelopoulos [23] examined the shrinkage strains in a cured acrylic photopolymer resin produced through SLA. Kechagias et al. [24] conducted an in-depth analysis of the relevant literature to study the effect of printing parameters on the FFF process’s dimensional accuracy and surface quality. Kantaros et al. [25] explored the residual strains in ABS parts manufactured by FDM through the use of the fiber Bragg grating method. Pourali applied a thermal modeling approach to material extrusion AM and found that higher printing and bed temperatures can improve mechanical properties. [26]. Casavola et al. [27] measured the residual stress in ABS FDM parts through a combination of the hole-drilling method and electronic speckle pattern interferometry. Due to the extensive number of printing variables involved in FFF, optimizing the printing conditions for each material using experimental methods can be challenging and labor-intensive. Thus, simulation and modeling approaches can be employed to efficiently assess the impact of processing conditions on the printed part. Numerical techniques also offer the advantage of examining the effects of these processing conditions on the crystallization kinetics and thermomechanical behavior of the printed polymer. Using simulation and modeling to optimize FFF printing conditions provides a more efficient and cost-effective way to study the various factors affecting the final product’s quality. By leveraging these techniques, researchers can accurately predict the printed polymer’s behavior under different processing conditions, allowing them to identify the optimal conditions for each material. This information is critical for improving the quality and performance of additively manufactured parts and increasing the range of materials that can be used in the FFF process.

Over the past few years, numerous studies have focused on predicting the mechanical behavior of 3D-printed parts using FFF. For instance, Croccolo et al. [28] created a mathematical prototype to estimate parts’ stiffness and tensile strength using FDM technology and confirmed the prototype through experimental tests. Domingo-Espin et al. [29] used FEA to examine the mechanical properties of FDM polycarbonate (PC) samples. They developed a model to simulate the behavior of FDM parts by comparing the findings of the mathematical model with experimental testing. They found that FEA isotropic model may provide better results than an orthotropic model in the elastic region. Zhou et al. [30] modeled the temperature analysis in the FDM method using the ANSYS software program to determine the temperature evolution at various times during the printing process. The analysis of prototypes manufactured using ABS filaments has shown that the choice of the modeling method significantly impacts the material’s thermal evolution by altering its thermal properties. Costa et al. [31] analyzed the heat dissipation and deformation of an FDM sample during the manufacturing process, considering a 3D extruded filament for the convection and radiation phenomena. They reported the temperature evolution in different parts of the specimen. Cattenone et al. [32] investigated the effect of various parameters on the mechanical performance of 3D-printed parts using the ABAQUS simulation software. They specifically analyzed the impact of factors such as mesh size, time-step size, and meshing strategy on the simulation results and experimentally validated them. The conducted research concluded that the time step significantly impacts the local temperature distribution during the 3D printing process, but it has a minor effect on the mechanical analysis results. The meshing strategy used in the simulation is crucial for accurately reproducing the real printing process. For smaller models, a finer meshing strategy is recommended to examine local effects, whereas, for larger models, a coarser meshing strategy is appropriate as local effects are negligible. Macedo and Ferreira [33] developed a simulation model for the FDM process that can calculate the temperature and stress during filament extrusion. The results of the study showed that printing without a heated bed resulted in higher stresses due to the rapid temperature changes that occurred during the process. Xia et al. [34] made a significant contribution toward the development of a numerical simulation methodology for the FDM process. Tests were conducted on the PLA polymer to examine its physical properties, including density, viscosity, thermal conductivity, and specific heat capacity. The outcomes of these simulations proved to be highly effective in the modeling of the FDM process. Zhang et al. [35] utilized an FEA mod incorporating element activations to analyze the sample’s thermal and mechanical behavior and calculate the residual stress. In addition to these analyses, the 3D model was also utilized to optimize the printing process by investigating the effects of various process parameters on part warpage and distortions. Similarly, Bertevas et al. [36] conducted a numerical investigation into the FDM 3D printing of fiber-reinforced polymer composites, as described in their publication. Their approach involved using the classical microstructure-based fiber suspension model, which was implemented through the smoothed particle hydrodynamics (SPH) method. By employing this methodology, they were able to analyze the influence of various factors, such as fiber distributions, aspect ratio, and orientations, on the FDM printing process. Their work contributes to the further understanding of the behavior of fiber-reinforced polymer composites during the FDM process and may help improve the quality and performance of such materials in the future. H. Alzyod and P Ficzere [37] employed Digimat-AM FEA to investigate the impact of five printing parameters on the warpage deformation of polyamide 12 (PA12) using FFF. They found that the bed temperature was the most significant parameter affecting the warpage deformation, with about 81% of the contribution. Their results agreed with experimental studies in the literature [38,39,40]. This study makes several novel contributions to the field of 3D printing. Firstly, it fills a significant gap in the existing literature by investigating the material-dependent effects of multiple printing parameters on residual stress and warpage deformation. While previous studies have explored the impact of individual parameters on these factors, a comprehensive analysis considering multiple parameters and materials is lacking. Secondly, by examining three different materials, this research expands the understanding of different material behaviors during 3D printing. Lastly, the use of the FEA and Taguchi L27 array enables a detailed simulation of the printing process, providing valuable insights into the underlying mechanisms that contribute to residual stress and warpage deformation and can help identify the most influential parameters that affect the quality of the final product.

The investigation is organized as follows. Firstly, the simulation procedure for FFF is explained in detail in Section 2 of the paper, including the used materials, FEA simulations, and the use of the Taguchi L27 array to evaluate the impact of the printing parameters on the residual stress and warpage deformation of the 3D-printed parts. The results and the statistical analysis are presented in Section 3, ensuring the normal distribution of the results. Section 4 discusses the results, highlighting the material-dependent effect of the printing parameters on each material’s residual stress and warpage deformation and how the relationship between the printing parameters and these factors varies depending on the material properties. Insights and observations from the study are also discussed, including the identification of the most influential printing parameters on the quality of the 3D-printed parts and the implications of the findings for optimizing the printing parameters for each material to minimize residual stress and warpage deformation. Finally, the key conclusions of the investigation are summarized in the final section, emphasizing the importance of considering the material properties when selecting the optimal printing parameters for 3D printing.

## 2. Numerical Process

### 2.1. Materials Selection and Used Samples

Three different materials were used in this study, namely PEI, ABS, and PA6. The materials’ properties data were selected from e-Xstream, which is part of Hexagon’s Manufacturing Intelligence division (MSC), and Table 1 illustrates the properties of these materials [41]. E-Xstream obtained these data through a combination of experimental testing, theoretical models, and data from reputable sources. These data are readable-only; users cannot modify or delete any existing values or import new ones [41]. Therefore, these data can be considered accurate and reliable. The filaments were unfilled, amorphous (PEI, ABS) and semi-crystalline (PA6), and had an isotropic coefficient of thermal expansion (CTE). A dogbone model was designed according to ASTM D638 using SolidWorks 2023 software (SolidWorks Corporation, Waltham, MA, USA) [42]. The model was then transferred to Ultimaker Cura 5.2.2 software program (Ultimaker B.V, Geldermalsen, Netherlands), which is compatible with the simulation software, to slice the part and convert it to G-code. The Taguchi fractional factorial method L27 was implemented using Minitab 2022 software (Minitab Ltd, Coventry, UK) to select the parameters or the factors and their levels for each run, in which a total of 81 runs were conducted to analyze the effect of the above six common printing parameters on the residual stress and warpage deformation of 3D-printed test parts made of three different materials: PEI, ABS, and PA6. The selection of PEI, ABS, and PA6 as the materials for this study was influenced by several factors. PEI is a high-performance engineering plastic and is widely utilized in the transportation industry due to its exceptional strength-to-weight ratio and low smoke evolution and toxicity. It is commonly used to create high-strength structural components and interior aircraft cabin parts [43]. PEI’s trade name is ULTEM™ 9085. During 3D printing, PEI requires high extrusion and bed temperatures to ensure proper bonding between layers. Additionally, PEI’s low density and low toxicity properties make it an attractive option for manufacturing aircraft cabin parts [44]. ABS is a commonly used thermoplastic polymer that is suitable for FFF 3D printing. One of the key advantages of using ABS for 3D printing is its toughness, making it an excellent choice for parts that require durability and impact resistance [14]. ABS is also a lightweight material, making it suitable for applications where weight is a critical factor. PA6 can be considered a suitable filament option due to its high toughness and impact resistance. However, it is essential to note that PA6 is highly sensitive to moisture and can warp similarly to ABS [45]. As with other FDM filaments, PA6 is also hygroscopic, meaning it can absorb moisture from the air. This moisture absorption can lead to a degradation of the filament’s properties, which can result in a degradation of the part’s characteristics [46]. All the specimens were sliced in an X-axis orientation and printed in the center of the printing bedplate.

### 2.2. Printing Parameter Selection

The selected parameters for this study are printing temperature, printing speed, bed temperature, infill density, layer thickness, and infill pattern. These parameters are described as follows:

- Printing temperature: This refers to the temperature of the 3D printer’s extruder nozzle, which melts the filament as it is deposited layer by layer to create the 3D-printed object. The temperature affects the viscosity of the filament, which in turn affects the flow rate and the bonding between layers.
- Printing speed: This parameter refers to the rate at which the printer head moves along the X–Y plane. Printing speed has an impact on the cooling time of each layer, which affects the strength of the bonds between layers.
- Bed temperature: This parameter refers to the temperature of the 3D printer’s build platform, which can be heated to promote a better adhesion between the printed object and the build surface. The temperature also affects the rate of the cooling of the bottom layer.
- Infill density: This refers to the amount of material that is used to fill the internal space of the 3D-printed object. Increasing the infill density can improve the strength and rigidity of the printed object, but it also increases the time and cost required to print the object.
- Layer thickness: This parameter refers to the height of each layer of the 3D-printed object. A thinner layer height produces a smoother surface finish, while a thicker layer height can reduce printing time.
- Infill pattern: This parameter refers to the shape and structure of the infill material within the printed object. Different infill patterns have different strengths and can affect the structural integrity of the 3D-printed object.

The selection of printing speed, infill density, layer thickness, and infill pattern was made according to the importance of their influence on the mechanical properties of FFF parts [24]. Nozzle and bed temperature parameters were selected based on limited studies and investigation of these parameters in the literature [47].

### 2.3. Taguchi Fractional Factorial Design

The Taguchi method is a statistical technique that aims to improve a product’s or process’s quality by optimizing design parameters. It utilizes an orthogonal array (OA) design to reduce the number of experiments needed to evaluate the impact of design parameters on the response, resulting in an efficient exploration of the design space [48]. This method can be applied to optimize the process parameters of FFF, reducing the time and cost required for the optimization process. The OA design allows for a reduced number of experiments while still providing valuable information on the impact of each parameter. The Taguchi method also employs the signal-to-noise (S/N) ratio to assess the quality of the FFF output, including dimensional accuracy, surface finish, and mechanical properties. The S/N ratio serves as a performance measure to optimize the process parameters by maximizing it, resulting in an improved quality and cost-effectiveness. The Taguchi method employs different types of responses or quality characteristics, such as ‘smaller is better’, ‘larger is better’, and ‘nominal is better’, to evaluate the system’s performance or process under study. ‘Smaller is better’ is used when a lower value of the output parameter is desirable. Examples include defects, errors, or manufacturing variations that should be minimized. ‘Larger is better’ is used when a higher value of the output parameter is desirable. Examples include strength, durability, or speed that should be maximized. ‘Nominal is better’ is used when a specific target value of the output parameter is desirable. Examples include the desired dimensions or tolerances of a part that must be achieved. In the Taguchi method, the chosen response type determines the choice of the signal-to-noise (S/N) ratio used to evaluate the performance of the system or process. For example, for the ‘smaller is better’ response, the Taguchi method uses the ratio of the mean of the signal (the desired output) to the standard deviation of the noise (the undesirable variation). Similarly, for the ‘larger is better’ response, the Taguchi method uses the ratio of the mean of the signal to the standard deviation of the total variation. For the ‘nominal is better’ response, the Taguchi method uses the percentage of parts that meet the target value. By selecting the appropriate type of response and corresponding S/N ratio, the Taguchi method allows the optimization of the process parameters to achieve the desired level of performance in the system or process. In this work, ‘smaller is better’ is selected to minimize the residual stress and warpage deformation of printed parts, and Equation (1) illustrates how to calculate the *S/N* ration [49]:

(1) $$S/N=\left(-10\right)\times \log_{10}\left(\frac{1}{n}\right)\sum_{i=1}^{n}y_{i}^{2}$$

where, *n* represents the number of runs and *y_i_* represents the response for a specific factor-level pattern.

The selected process parameters and their levels are presented in Table 2. The selection of these levels was chosen by taking into account the literature studies, the technical datasheet provided by filament manufacturers, as illustrated in Table 1, and the slicer software and printer associated with the simulation.

### 2.4. Simulation Procedure

The utilization of the numerical simulation for the FFF technique helps to offer significant benefits. The key benefits are the potential to save both the cost and time that would be required for physical prototyping. A numerical solution can be used to foresee a part’s final characteristics, including warpage deflection and mechanical characteristics, without requiring multiple physical prototypes’ printing and testing.

**Figure 2:**
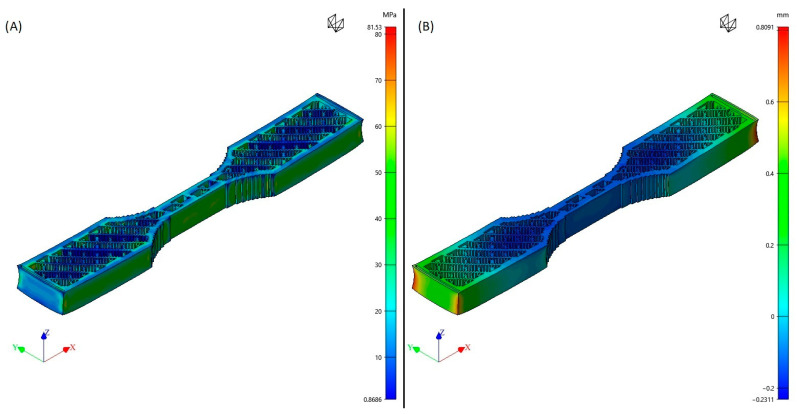
Numerical results of the DigimatAM simulation from run 13 of PA6 material: (**A**) residual stress, (**B**) warpage deformation.

Using numerical solutions in FFF can significantly save cost and time during the investigation and development procedure. These solutions allow for the optimization of the printing process by predicting the influence of several printing factors, including extruding temperature, chamber temperature, extruding speed, and layer height, on the final properties of a printed part. This optimization helps in identifying the ideal printing parameters and enhances the printed parts’ quality. Moreover, the numerical solution could help in predicting the mechanical characteristics of FFF parts, like ultimate strength and toughness, depending on material characteristics like density, Poisson ratio, and the modulus of elasticity and printing parameters, which can assist in selecting the proper materials and printing parameters for a specific application or implementation. In this study, computer-aided engineering (CAE) simulations were utilized with Digimat-AM 2021 software (e-Xstream engineering, Hautcharage, Luxembourg). Digimat-AM is software specializing in process simulation for the AM of polymer and composite materials. This tool enables process engineers to anticipate various factors such as warpage, residual stresses, temperature history, and microstructure changes that a printed part may undergo based on the chosen process parameters, printing strategy, and material selection. By utilizing Digimat-AM simulations, the printer setup can be optimized before physically printing the part. This can be accomplished by determining the appropriate warpage compensation for the designed geometry. The software offers an efficient and straightforward workflow that starts with defining the printing project, specifying manufacturing parameters, setting up the simulation, and finally, post-processing the results. This process allows for the efficient optimization of printing setup prior to physical printing, which can lead to reduced costs and time savings in the research and development process. Digimat-AM offers a guided six-step workflow to optimize the AM process of polymers and composites. The printing project is the initial step that enables the selection of the desired printing process, a specific printer, and the type of analysis on the printing process, whether thermal or warpage. In this study, the FFF process, a generic printer with dimensions of 250 mm × 250 mm × 210 mm and warpage analysis were chosen. Component is the second step, which involves importing the component geometry, which can be obtained as a .stl file, and selecting the material that will be used in printing, such as an unfilled or reinforced polymer. Manufacturing is the third step that allows for the description of how the component is manufactured, including various inputs such as positioning, toolpath (imported as a G-code file from the slicer software), and the order of manufacturing steps. In this study, the part was positioned in the center of the bedplate, and the manufacturing steps were ordered as printing, holding, cooling, and support removal. The fourth step is solver, which translates the previous settings into an actual FEA simulation. The voxel meshing of the geometry is proposed, solution methods can be chosen, and material model parameters can be adjusted. Afterward, the job submission step follows suit. Once the simulation model is ready, it can be submitted and monitored until reaching job completion in the fifth step. Finally, the post-processing step provides all the functionalities required to post-process the simulation results, including the field visualization of deformation and residual stresses. Figure 2 illustrates those results from run 13 of the PA6 material.

## 3. Numerical Results and Statistical Analysis

Table A1, Table A2 and Table A3 illustrate the L27 array and the residual stress and warpage deformation for the PEI, ABS, and PA6 materials, respectively.

### Probability Plots and ANOVA

**Figure 3:**
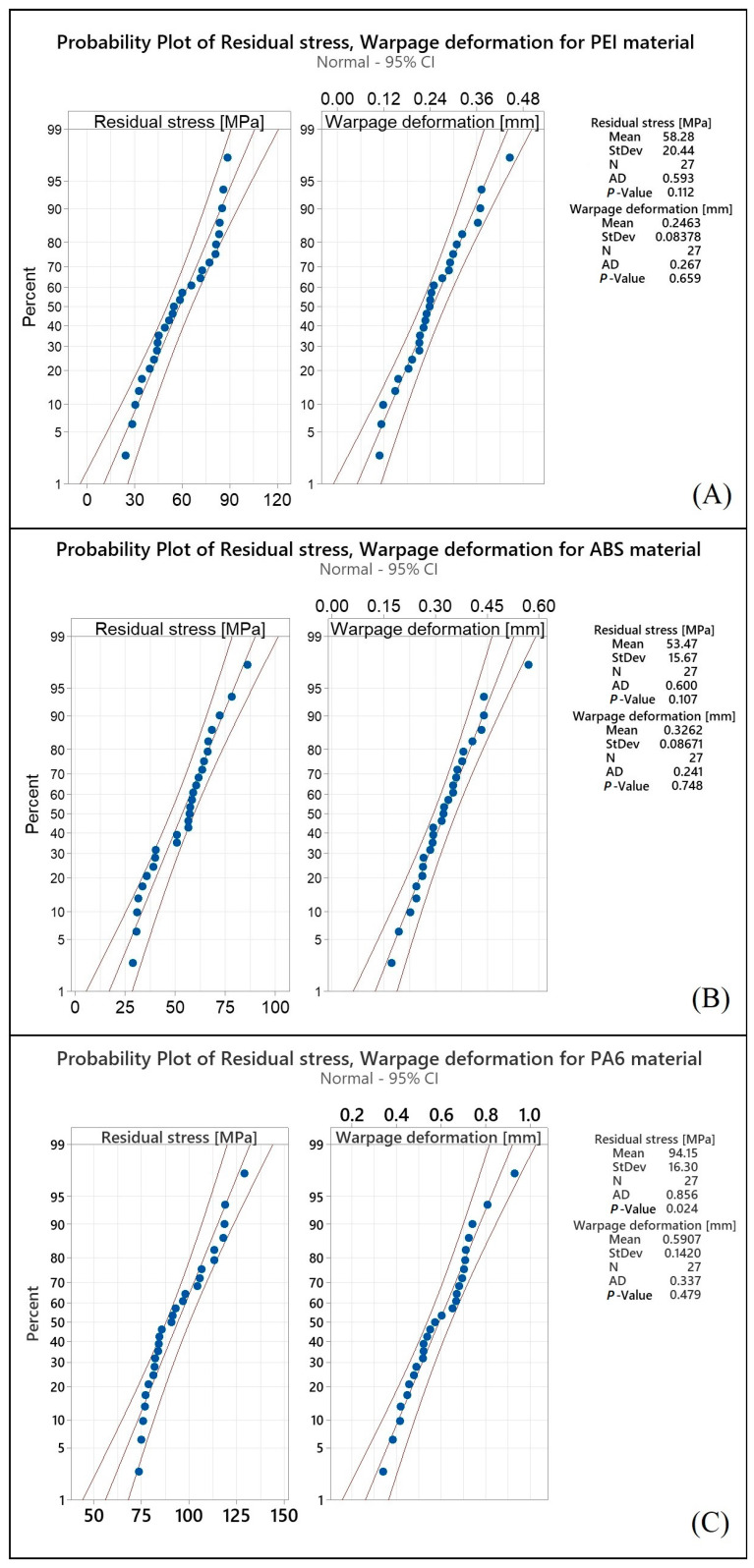
Probability plot of residual stress and warpage deformation for: (**A**) PEI, (**B**) ABS, and (**C**) PA6 materials.

Probability plots are a valuable tool for assessing the fit of a dataset to a particular probability distribution. Probability plots enable researchers to visualize the relationship between the observed values of a variable and the expected values under a theoretical distribution. This graphical representation can aid in identifying departures from the assumed distribution, which can have important implications for the validity of statistical tests and models. The Anderson–Darling (AD) test, a statistical test that can be used to test if a set of data is normally distributed, was utilized in this study to validate the normality assumption. Smaller AD test statistics and a larger p-value indicate a better fit of the data to a normal distribution [50]. As shown in Figure 3, the collected results for the residual stress and warpage deformation responses closely align with the fitted line, and the AD test statistics values are relatively small, while the p-values are greater than the commonly used significance level of 0.05. Based on these results, it is reasonable to assume that the collected data follow a normal distribution. Consequently, additional analyses and optimization can be conducted on the data with confidence in the normality assumption. The ANOVA statistical technique is widely used in many research fields to determine the significance of input parameters on various outcomes [51]. By performing an ANOVA with a 95% confidence interval, the results obtained in this study can be considered to be reliable and accurate. The significance of the parameters is determined by the p-value, which represents the probability of obtaining the observed results by chance. In this study, a p-value less than 0.05 was used to indicate the significance of the parameter. The probability plot and ANOVA analysis were carried out using Minitab 2022 software.

## 4. Discussion

### 4.1. ANOVA and Mean Effect Plot (MEP) for Residual Stress

**Figure 4:**
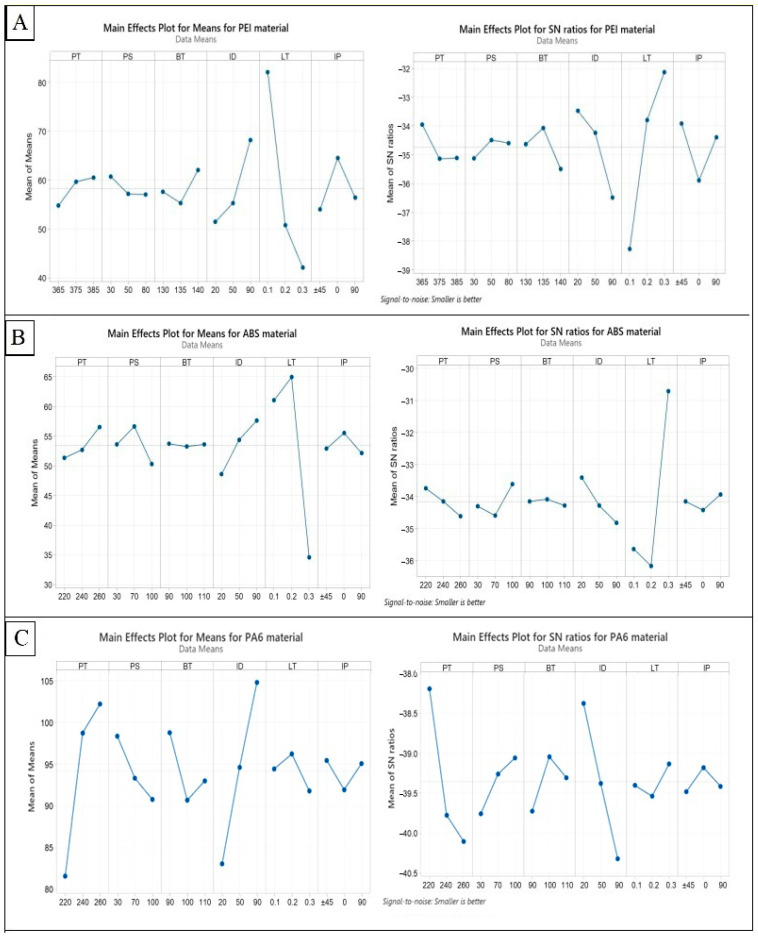
Main effect plot of means and S/N ratios for residual stress: (**A**) PEI, (**B**) ABS, and (**C**) PA6.

The results of the ANOVA for residual stress were obtained for three different materials (PEI, ABS, and PA6), and the contribution percentage for each parameter is presented in Table 3, and Figure 4 depicts the MEP and S/N ratios for the residual stress response. Regarding the PEI material, the largest contribution to residual stress comes from layer thickness (62.94%), followed by infill density (15.25%) and infill pattern (6.66%). The remaining parameters have relatively smaller contributions with no significance, with printing temperature, bed temperature, and printing speed having contributions of 2.88%, 3.23%, and 0.73%, respectively. These results suggest that controlling layer thickness and infill density can significantly impact reducing residual stress in PEI 3D-printed parts. Printing temperature results agreed with Ding et al. [43] within the same printing temperature range and how the printing temperature increase negatively affects the printed parts’ mechanical properties. As shown in Figure 4A, infill density exhibits a positive correlation with mean residual stress, indicating that a higher value of this parameter is associated with an increased residual stress. Higher infill densities result in a denser internal structure with reduced void spaces, thus increasing material interactions and residual stress during the printing process. Conversely, layer thickness demonstrates a negative correlation with mean residual stress. Thinner layers allow for faster cooling and solidification, reducing the buildup of internal stresses. For ABS, the largest contribution to residual stress is from layer thickness (83.40%), followed by infill density (4.64%) and printing speed (2.32%). The rest parameters do not influence the residual stress value, with contributions of less than 5% for all. Even the layer thickness was the most significant parameter, and it has a non-linear relation with the residual stress, see Figure 4B. This can be explained by the complex interplay between heat transfer, thermal expansion, and contraction during the cooling phase of ABS material. This significant contribution of the layer thickness was in very close agreement with a previous experimental study [52], which reported that the layer thickness contributed about 85% to the dimensional accuracy of printed ABS parts. Higher residual stress levels in a printed part can lead to dimensional inaccuracies. This is because residual stress can cause the part to deviate from the intended dimensions. The magnitude and distribution of residual stress within the printed part can affect its overall shape, size, and dimensional stability. Moreover, Daly M et al. [53] confirmed that the residual stress decreases as the printing speed increases for FFF ABS samples. Similarly, Zhang et al. [54] investigated the effect of infill pattern and printing speed on the residual stress of ABS FFF printed samples. They found that the residual stress positively correlated with the printing speed. Furthermore, when considering the infill pattern, the results revealed that the 0° infill pattern exhibited the highest residual stress, followed by the 90° pattern, while the minimum residual stress was obtained in the ±45° pattern. These findings verified the outcomes of our investigation.

Finally, in PA6, the largest contribution to residual stress is from printing temperature (33.81%), followed by infill density (30.73%). Printing speed, bed temperature, layer thickness, and infill pattern have relatively smaller contributions, with contributions of 4.19%, 3.82%, 1.37%, and 0.82%, respectively. The printing temperature and infill density positively correlate with the mean residual stress, and Figure 4C depicts this. This can be attributed to the fact that higher temperatures promote a greater thermal expansion and induce higher thermal stresses within the printed part. As a result, the material experiences increased residual stress levels, which can negatively influence the elongation properties of PA6 material. Residual stress can introduce additional internal forces within the material, which can affect its deformation behavior under tensile loading. This can result in a reduction in elongation percentage and potentially lead to premature failure or decreased ductility; and Ali, L. Feroz et al. [55] demonstrated the relationship between increasing infill density and the corresponding decrease in the elongation of PA6. The findings indicated that as the infill density increased, the elongation of PA6 decreased. This suggests that higher infill densities negatively impact the material’s ability to undergo plastic deformation and elongation before failure.

Based on the ANOVA results of the three materials, it is clear that the parameters that contribute the most to residual stress are different for each material. For instance, in PEI and ABS, the layer thickness had the largest contribution to residual stress, while in PA6, printing temperature had the most significant impact. This indicates that optimizing printing parameters to reduce residual stress requires a material-specific approach. In other words, the same printing parameters that work well for one material may not work as effectively for another. Therefore, it is crucial to conduct material-specific analyses when selecting and optimizing 3D printing parameters to achieve the desired quality of the 3D-printed part. This scientific explanation highlights the importance of material-specific analyses for optimizing 3D printing parameters, improving quality and reducing the residual stress of 3D-printed parts. By understanding the contribution of each parameter to residual stress in each material, researchers can develop a more effective approach to optimize printing parameters for specific materials, leading to better results and a higher-quality final product.

### 4.2. ANOVA and Mean Effect Plot (MEP) for Warpage Deformation

**Figure 5:**
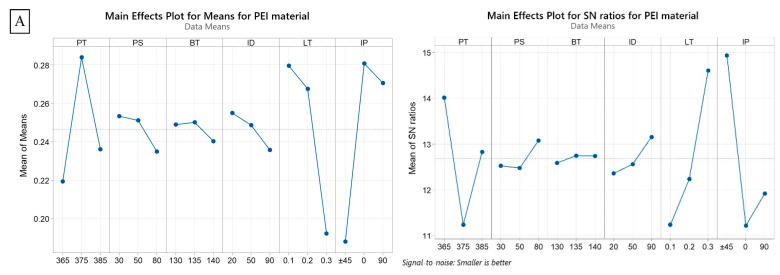

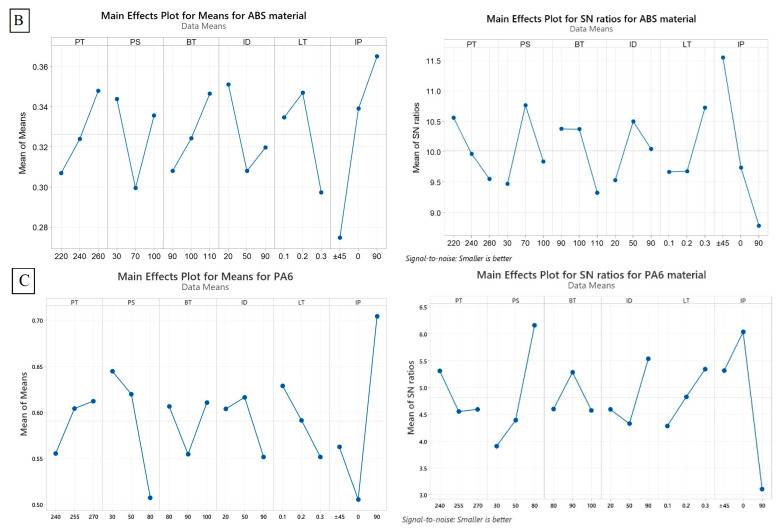
Main effect plot of means and S/N ratios for warpage deformation: (**A**) PEI, (**B**) ABS, and (**C**) PA6.

Table 4 shows the results of the ANOVA for the warpage deformation response obtained for the three different materials (PEI, ABS, and PA6) and the contribution percentage of each process parameter; and Figure 5 illustrates MEP and S/N ratios for the warpage deformation response. The contributions are expressed as a percentage of the total variation in the warpage deformation. For PEI, the parameters with significant contributions to warpage deformation are infill pattern and layer thickness, with contributions of 27.05% and 20.73%, respectively. Printing temperature has a good contribution, even if it is not significant, with a contribution of 13.7%. On the other hand, printing speed, bed temperature, and infill density have no significant contributions to warpage deformation for PEI, with less than 2% of the contribution for each one. The data presented in Figure 5A reveal that the mean warpage deformation varies depending on the infill pattern used. Specifically, the infill pattern at ±45° exhibits the lowest mean warpage deformation, followed by the 90° pattern. On the other hand, the highest mean warpage deformation is observed when using the 0° infill pattern. Thus, the influence of the infill pattern on the properties of PEI can be observed from the studies conducted by Kaplun et al. [56] and Yogeshwaran [57]. These studies specifically highlighted how the choice of infill pattern and orientation could significantly impact the mechanical properties of PEI parts manufactured using FDM. For ABS, since it is most sensitive regarding warpage deformation due to its high glass transition temperature [58], there is no individual parameter with a significant contribution to warpage deformation. Therefore, controlling the warpage deformation of ABS can be influenced by more than one parameter or interaction between parameters. Infill pattern was the largest contributor, with a contribution of 25.30%. Printing temperature, printing speed, bed temperature, infill density, and layer thickness have no significant contributions to warpage deformation for ABS, with each parameter accounting for less than 5.69% of the variation. The specific values depicted in Figure 5B indicate that the infill pattern at ±45° results in the lowest mean warpage deformation, which aligns with the previous experimental study [54], followed by the 0° pattern, while the highest mean warpage deformation is observed with the 90° pattern. It is worth mentioning that the printing speed has a varying impact on warpage deformation, as observed in another study [53]. Initially, increasing the printing speed can lead to a decrease in warpage, but beyond a certain point, further speed increases can increase warpage deformation.

For PA6, the parameters significantly contributing to warpage deformation are infill pattern and printing speed, with contributions of 35.38% and 21.19%, respectively. Printing temperature, bed temperature, infill density, and layer thickness have no significant contributions to warpage deformation for PA6. Figure 5C shows a negative correlation between printing speed and warpage deformation, suggesting that higher printing speeds tend to result in a reduced warpage deformation in PA6. Higher printing speeds allow for a better cooling and solidification of the material, minimizing the occurrence of internal stresses that can lead to warping. For the infill pattern, the mean values of warpage deformation vary across the different infill pattern levels. The results indicate that the 90° pattern results in the highest mean warpage deformation, followed by the ±45° pattern, and the lowest mean warpage deformation is observed with the 0° pattern. This suggests that the infill pattern plays a role in influencing warpage deformation in PA6. Different infill patterns introduce variations in the internal structure of the printed part. In a comprehensive study conducted by Peng Xingshuang et al. [59], the effect of printing parameters on the mechanical properties of PA6 was thoroughly investigated. Notably, the study revealed that the infill pattern significantly determined the tensile strength and Young’s modulus of the 3D-printed components. Specifically, it was observed that the 90° pattern exhibited the lowest values for both tensile strength and Young’s modulus, followed by the ±45° pattern. Conversely, the highest values for these mechanical properties were consistently observed with the 0° pattern. These findings shed light on the critical influence of warping on the mechanical properties of 3D-printed components. Warping introduces internal stresses and structural irregularities within the printed parts, consequently reducing their strength and stiffness. Due to the semi-crystalline nature of PA6, the printing process poses significant challenges. The high degree of crystallinity often leads to issues such as pronounced shrinkage, interlayer delamination, the presence of warping, and porosity, and consequently affects both the dimensional stability and mechanical performance of the printed part [60,61]. These results indicate that specific parameters play a more significant role in warpage deformation for each material than others. It is, therefore, important to conduct material-specific analyses when optimizing 3D printing parameters to minimize warpage deformation and improve the quality of 3D-printed parts.

## 5. Conclusions

The study investigated the effect of common 3D printing parameters on residual stress and warpage deformation in three different materials (PEI, ABS, and PA6) using finite element analysis (FEA). ANOVA statistical technique was used to determine the significance of input parameters on various outcomes with a 95% confidence interval, and a p-value less than 0.05 was used to indicate the significance of the parameter. Results showed that material-specific analyses are required when selecting and optimizing 3D printing parameters to reduce residual stress and warpage deformation and achieve the desired quality of the 3D-printed part, and we can conclude that:

- The importance of each parameter in relation to residual stress varies depending on the material being printed. This indicates that all investigated parameters are material-dependent to some extent.
- Layer thickness is a consistently important parameter affecting the residual stress value for all three materials, suggesting that it is relatively less sensitive to material differences.
- Infill density demonstrates the varying levels of importance among materials influencing the residual stress. PA6 shows the highest contribution (30.73%), followed by PEI (15.25%) and ABS (4.64%). This implies that infill density is more sensitive to material differences.
- Printing temperature significantly contributes to reducing residual stress in PA6 (33.81%), while it does not significantly impact PEI and ABS. This suggests that printing temperature is more material-dependent.
- The infill pattern parameter demonstrates the highest contribution to reducing warpage deformation across all three materials, namely PEI, ABS, and PA6. This observation highlights its significance in minimizing warpage, irrespective of material variations.
- In terms of printing temperature, it exerts a relatively low impact on warpage deformation for PA6 (2.74%) while having a moderate effect on ABS (3.3%) and high PEI (13.4%).
- Printing speed exhibits a minor influence on mitigating warpage deformation for PEI (0.77%) and ABS (5.69%), but it significantly affects PA6 with a substantial impact of 21.19%.
- Bed temperature has a minimal effect on warpage deformation for PEI (0.06%), a relatively higher impact for ABS (4.69%), and a medium influence on PA6 (2.45%).
- Infill density demonstrates a limited influence on warpage deformation for PEI (1.18%), while it exerts a considerable effect on PA6 (6.18%). For ABS, its impact falls within the moderate range (3%).
- Layer thickness plays a crucial role in warpage deformation, yielding a substantial impact for PEI (20.73%) and a moderate effect for ABS (4.74%) and PA6 (4.29%).
- The limited research available on the material-specific effects of PEI in 3D printing calls for future investigations to explore its unique characteristics, such as thermal and mechanical properties, and their impact on residual stress and warpage deformation.

The study highlights the importance of conducting material-specific analyses when optimizing 3D printing parameters to achieve the desired quality and minimize residual stress and warpage deformation. The findings provide useful insights for researchers and practitioners in the field of 3D printing, emphasizing the need for material-specific approaches in developing effective printing parameter optimization strategies.

## Figures and Tables

**Table 1:** Properties of used materials [41].

Properties	Material		
	PEI	ABS	PA6
Filament diameter (mm)	1.75	1.75	1.75
Density (g/cm^3^)	1.34	1.04	1.14
Poisson ratio	0.35	0.36	0.37
Printing temperature (°C)	365–385	220–260	240–270
Bed temperature (°C)	130–140	90–110	80–100
Printing speed (mm/s)	30–80	30–100	30–80
Strength (MPa)	54	43	80
Elasticity modulus (MPa)	2050	1750	3300

**Table 2:** FFF process parameters and corresponding levels.

Parameter	Units	Material	Levels		
			1	2	3
Printing temperature (PT)	(°C)	PEI/ABS/PA6	365/220/240	375/240/255	385/260/270
Printing speed (PS)	(mm/s)	PEI/ABS/PA6	30	50/70/50	80/100/80
Bed temperature (BT)	(°C)	PEI/ABS/PA6	130/90/80	135/100/90	140/110/100
Infill density (ID)	(%)		Low (20)	Medium (50)	High (90)
Layer thickness (LT)	(mm)		0.2	0.3	0.4
Infill pattern (IP)			0°	±45°	90°

**Table 3:** ANOVA for residual stress response.

Material	Source	DF	Seq SS	Adj SS	Adj MS	F	P	% Contribution
PEI	PT	2.00	8.29	8.29	4.14	2.43	0.12	2.88
	PS	2.00	2.10	2.10	1.05	0.62	0.55	0.73
	BT	2.00	9.28	9.28	4.64	2.72	0.10	3.23
	ID	2.00	43.85	43.85	21.93	12.85	0.00	15.25
	LT	2.00	180.99	180.99	90.50	53.03	0.00	62.94
	IP	2.00	19.15	19.15	9.57	5.61	0.02	6.66
	Error	14.00	23.89	23.89	1.71			8.31
	Total	26.00	287.54					100.00
ABS	PT	2.00	3.42	3.42	1.71	1.69	0.22	1.75
	PS	2.00	4.54	4.54	2.27	2.24	0.14	2.32
	BT	2.00	0.18	0.18	0.09	0.09	0.92	0.09
	ID	2.00	9.09	9.09	4.54	4.49	0.03	4.64
	LT	2.00	163.24	163.24	81.62	80.62	0.00	83.40
	IP	2.00	1.10	1.10	0.55	0.54	0.59	0.56
	Error	14.00	14.17	14.17	1.01			7.24
	Total	26.00	195.73					100.00
PA6	PT	2.00	18.79	18.79	9.40	9.37	0.00	33.81
	PS	2.00	2.33	2.33	1.16	1.16	0.34	4.19
	BT	2.00	2.12	2.12	1.06	1.06	0.37	3.82
	ID	2.00	17.08	17.08	8.54	8.51	0.00	30.73
	LT	2.00	0.76	0.76	0.38	0.38	0.69	1.37
	IP	2.00	0.46	0.46	0.23	0.23	0.80	0.82
	Error	14.00	14.04	14.04	1.00			25.26
	Total	26.00	55.58					100.00

**Table 4:** ANOVA for warpage deformation response.

Material	Source	DF	Seq SS	Adj SS	Adj MS	F	P	% Contribution
PEI	PT	2	34.67	34.67	17.34	2.55	0.11	13.40
	PS	2	1.99	1.99	0.99	0.15	0.87	0.77
	BT	2	0.14	0.14	0.07	0.01	0.99	0.06
	ID	2	3.05	3.05	1.52	0.22	0.80	1.18
	LT	2	53.65	53.65	26.83	3.94	0.04	20.73
	IP	2	69.99	69.99	35.00	5.14	0.02	27.05
	Error	14	95.28	95.28	6.81			36.82
	Total	26	258.78					100.00
ABS	PT	2.00	4.62	4.62	2.31	0.43	0.66	3.30
	PS	2.00	7.96	7.96	3.98	0.75	0.49	5.69
	BT	2.00	6.56	6.56	3.28	0.62	0.55	4.69
	ID	2.00	4.20	4.20	2.10	0.39	0.68	3.00
	LT	2.00	6.63	6.63	3.32	0.62	0.55	4.74
	IP	2.00	35.40	35.40	17.70	3.32	0.07	25.30
	Error	14.00	74.54	74.54	5.32			53.28
	Total	26.00	139.91					100.00
PA6	PT	2.00	3.26	3.26	1.63	0.69	0.52	2.74
	PS	2.00	25.16	25.16	12.58	5.34	0.02	21.19
	BT	2.00	2.91	2.91	1.46	0.62	0.55	2.45
	ID	2.00	7.33	7.33	3.67	1.56	0.25	6.18
	LT	2.00	5.09	5.09	2.54	1.08	0.37	4.29
	IP	2.00	42.00	42.00	21.00	8.92	0.00	35.38
	Error	14.00	32.97	32.97	2.36			27.77
	Total	26.00	118.72					100.00

## Data Availability

Data available on request.

## Appendix A

**Table A1:** Experimental design and results using L27 of PEI material.

Run	PT (°C)	PS (mm/s)	BT (°C)	ID (%)	LT (mm)	IP (°)	Residual Stress (MPa)	Warpage Deformation (mm)
1	365	30	130	20	0.1	0	83.53	0.37
2	365	30	130	20	0.2	±45	34.72	0.12
3	365	30	130	20	0.3	90	30.57	0.22
4	365	50	135	50	0.1	0	81.12	0.36
5	365	50	135	50	0.2	±45	33.00	0.11
6	365	50	135	50	0.3	90	28.50	0.21
7	365	80	140	90	0.1	0	88.67	0.25
8	365	80	140	90	0.2	±45	54.11	0.21
9	365	80	140	90	0.3	90	58.49	0.11
10	375	30	135	90	0.1	±45	85.95	0.21
11	375	30	135	90	0.2	90	65.89	0.45
12	375	30	135	90	0.3	0	54.90	0.19
13	375	50	140	20	0.1	±45	81.58	0.31
14	375	50	140	20	0.2	90	39.69	0.29
15	375	50	140	20	0.3	0	44.68	0.27
16	375	80	130	50	0.1	±45	77.34	0.23
17	375	80	130	50	0.2	90	44.10	0.37
18	375	80	130	50	0.3	0	42.41	0.23
19	385	30	140	50	0.1	90	85.20	0.24
20	385	30	140	50	0.2	0	60.16	0.29
21	385	30	140	50	0.3	±45	45.31	0.18
22	385	50	130	90	0.1	90	83.48	0.30
23	385	50	130	90	0.2	0	72.74	0.24
24	385	50	130	90	0.3	±45	49.35	0.16
25	385	80	135	20	0.1	90	71.72	0.24
26	385	80	135	20	0.2	0	51.93	0.32
27	385	80	135	20	0.3	±45	24.39	0.15

**Table A2:** Experimental design and results using L27 of ABS material.

Run	PT (°C)	PS (mm/s)	BT (°C)	ID (%)	LT (mm)	IP (°)	Residual Stress (MPa)	Warpage Deformation (mm)
1	220	30	90	20	0.1	0	50.87	0.41
2	220	30	90	20	0.2	±45	57.39	0.23
3	220	30	90	20	0.3	90	31.54	0.36
4	220	70	100	50	0.1	0	78.04	0.24
5	220	70	100	50	0.2	±45	56.40	0.17
6	220	70	100	50	0.3	90	30.55	0.36
7	220	100	110	90	0.1	0	50.91	0.28
8	220	100	110	90	0.2	±45	72.24	0.32
9	220	100	110	90	0.3	90	33.59	0.38
10	240	30	100	90	0.1	±45	63.32	0.26
11	240	30	100	90	0.2	90	66.09	0.44
12	240	30	100	90	0.3	0	40.18	0.29
13	240	70	110	20	0.1	±45	56.53	0.44
14	240	70	110	20	0.2	90	60.51	0.32
15	240	70	110	20	0.3	0	35.75	0.26
16	240	100	90	50	0.1	±45	61.51	0.29
17	240	100	90	50	0.2	90	58.87	0.34
18	240	100	90	50	0.3	0	31.11	0.26
19	260	30	110	50	0.1	90	64.27	0.35
20	260	30	110	50	0.2	0	68.12	0.43
21	260	30	110	50	0.3	±45	40.14	0.32
22	260	70	90	90	0.1	90	66.35	0.35
23	260	70	90	90	0.2	0	86.09	0.29
24	260	70	90	90	0.3	±45	39.15	0.24
25	260	100	100	20	0.1	90	57.05	0.38
26	260	100	100	20	0.2	0	58.30	0.57
27	260	100	100	20	0.3	±45	28.84	0.19

**Table A3:** Experimental design and results using L27 of PA6 material.

Run	PT (°C)	PS (mm/s)	BT (°C)	ID (%)	LT (mm)	IP (°)	Residual Stress (MPa)	Warpage Deformation (mm)
1	240	30	80	20	0.1	0	77.43	0.54
2	240	30	80	20	0.2	±45	76.17	0.71
3	240	30	80	20	0.3	90	84.20	0.67
4	240	50	90	50	0.1	0	76.94	0.52
5	240	50	90	50	0.2	±45	82.38	0.55
6	240	50	90	50	0.3	90	73.70	0.65
7	240	80	100	90	0.1	0	96.94	0.42
8	240	80	100	90	0.2	±45	90.93	0.34
9	240	80	100	90	0.3	90	75.10	0.60
10	255	30	90	90	0.1	±45	119.00	0.49
11	255	30	90	90	0.2	90	104.60	0.74
12	255	30	90	90	0.3	0	106.70	0.52
13	255	50	100	20	0.1	±45	81.53	0.81
14	255	50	100	20	0.2	90	83.83	0.67
15	255	50	100	20	0.3	0	91.57	0.52
16	255	80	80	50	0.1	±45	93.24	0.57
17	255	80	80	50	0.2	90	129.20	0.70
18	255	80	80	50	0.3	0	78.97	0.42
19	270	30	100	50	0.1	90	105.60	0.70
20	270	30	100	50	0.2	0	98.26	0.71
21	270	30	100	50	0.3	±45	113.30	0.72
22	270	50	80	90	0.1	90	113.40	0.93
23	270	50	80	90	0.2	0	118.60	0.45
24	270	50	80	90	0.3	±45	118.00	0.48
25	270	80	90	20	0.1	90	85.95	0.68
26	270	80	90	20	0.2	0	82.04	0.46
27	270	80	90	20	0.3	±45	84.60	0.38
